# 

**Published:** 1892-06-18

**Authors:** 


### CONTENTS.

**Samaritans Ancient and Modern** - - - - - - 177

**Passing Topics.**—A Central Council - - - - - - 178

**The Doctor's Armchair.**—The Sincerity of Modern Medicine - 179

**The History and Capabilities of Herbal Simples.** By W. T. Fernie, M.D. XXXIX.—The Fig - - - - - 180

**Medical History from the Earliest Times.** By E. T. Withington, M.A., M.B.Oxon. XIII.—The Alexandrine Anatomists - - - - - - - - - - - - - 181

**Everybody's Page** - - - - - - - - - - - 182

**Annotations.**—Lord Tennyson's Appeal—The "Lancet's" Help—The Street Beggars of Charity - - - - - - - - 183

**Notes and News** - - - - - - - - - - - 184

**Around the Hospitals—Scraps and Gleanings** - - - 185

**The Practitioners' Mirror and Retrospect—**

Medicine.—The Position of the Medical Examiner in Life Assurance.—I. - - - - - - - - - - 186

Surgery.—Diagnosis in Stricture of the Œsophagus - - - 187

Practitioners' Bookshelf.—The Nine Circles—Lunacy Law - 188

New Drugs, Appliances, and Things Medical.—Marriott's Patent Lentiline Biscuits—Niemann's Carnine Syrup—Peterman's Cockroach and Beetle Food - - - - - - 188

**The Institutional Workshop—**

A Year's Work in the Hospitals and Medical Charities of London - - - - - - - - - - - 189

**The Story of the Insane from Year to Year** - - - - 192

**The Student of Medicine.**—Appointments—Vacancies—Athletics.

### EXTRA SUPPLEMENT.—THE NURSING MIRROR.

**En Passant.**—Edinburgh Royal Infirmary—Nurses' Co-operation—A Trained Nurse for Castor—Bedfordshire Nurses' Institute—Wants and Workers—Short Items—Edinburgh Lady Medical Students—Opening of New Wards at Cold Ash Cottage Hospital—Tea and Tea Drinking—A Warning to Nurses—Trains and Trained Nurses - - - - - - - - lxxix

**Ventilation, Disinfection, and Diet.** By P. Caldwell Smith, M.D. X.—Disinfection (*continued*) - - - - lxxx

**Where to Go—Presentations—Notes and Queries** - - lxxx

**Nursing Homes.** VI.—London Hospital - - - - - lxxxi

**For Reading to the Sick.**—Contentment - - - - - lxxxi

**Recent Advances in Abdominal Surgery: Sterilised Salt Solution—Nurses' Outfits** - - - - - - lxxxii

**Everybody's Opinion.**—Private Nurses and their Ways - lxxxiii

**Some Australian Experiences** - - - - - - - lxxxiv

**"What the 'Register of Trained Nurses' Really Is"** lxxxiv

**Four Months in a Hospital Wad.**—V. - - - - - lxxxv

**Off Duty.**—A Sheep in Wolf's Clothing - - - - - lxxxvi

